# CHADS_2_, CHA_2_DS_2_-VASc, ATRIA, and Essen stroke risk scores in stroke with atrial fibrillation: A nationwide multicenter registry study

**DOI:** 10.1097/MD.0000000000024000

**Published:** 2021-01-22

**Authors:** Inwu Yu, Tae-Jin Song, Bum Joon Kim, Sung Hyuk Heo, Jin-Man Jung, Kyung-Mi Oh, Chi Kyung Kim, Sungwook Yu, Kwang Yeol Park, Jeong-Min Kim, Jong-Ho Park, Jay Chol Choi, Man-Seok Park, Joon-Tae Kim, Yang-Ha Hwang, Jong-Won Chung, Oh Young Bang, Geong-Moon Kim, Yong-Jae Kim, Seonwoo Kim, Sook young Woo, Hyun Cho, Woo-Keun Seo

**Affiliations:** a Department of Neurology, Samsung Medical Center, Sungkyunkwan University School of Medicine; b Department of Neurology, Seoul Hospital Ewha Womans University College of Medicine; c Department of Neurology, Stroke Center, Asan Medical Center; d Department of Neurology, Kyung Hee University College of Medicine, Seoul; e Department of Neurology, Korea University Ansan Hospital, Korea University College of Medicine, Ansan, Kyungki-Do; f Department of Neurology, Korea University Guro Hospital; g Department of Neurology, Korea University Anam Hospital, Korea University College of Medicine; h Department of Neurology, Chung-Ang University College of Medicine; i Department of Neurology, Seoul National University Hospital, Seoul; j Department of Neurology, Myongji Hospital, Hanyang University College of Medicine, Goyang; k Department of Neurology, Jeju National University, Jeju; l Department of Neurology, Chonnam National University Hospital, Gwangju; m Department of Neurology, Cerebrovascular Center, Kyungpook National University School of Medicine and Hospital, Daegu; n Department of Neurology, Eunpyeong St. Mary's Hospital, the Catholic University of Korea; o Statistics and Data Center, Samsung Medical Center, Seoul, Republic of Korea.

**Keywords:** ATRIA score, atrial fibrillation, CHA_2_DS_2_-VASc score, CHADS_2_ score, secondary prevention, stroke

## Abstract

The performance of scoring systems for risk stratification in patients with atrial fibrillation (AF) was not validated well in patients with stroke. The purpose of this study was to evaluate whether the risk scoring systems predict vascular outcomes in stroke patients with AF.

Data were obtained from a nationwide multicenter registry for acute stroke with AF from January 1, 2013, to December 31, 2015. We investigated the predictive power of the CHADS_2_, CHA_2_DS_2_-VASc, ATRIA, and Essen stroke scores in stroke patients with AF. The subjects were further stratified into groups according to treatment with or without oral anticoagulants (OACs).

A total of 3112 stroke with AF subjects were included. The rate of recurrent ischemic stroke and any stroke were not associated with the CHADS_2_, CHA_2_DS_2_-VASc, ATRIA, and Essen stroke risk scores. The risks of death and major adverse cerebrovascular and cardiovascular events (MACEs) increased sequentially with the increase of each risk score in OAC group. (the range of C-index 0.544–0.558 for recurrent ischemic stroke; 0.523–0.537 for any stroke; 0.580–0.597 for death; 0.564–0.583 for MACEs). However, in the group treated with OACs, all risk scores were significantly associated with the risk of MACEs. The C-statistics of the 4 scoring systems were 0.544 to 0.558, 0.523 to 0.537, 0.580 to 0.597, 0.564 to 0.583, respectively, for recurrent ischemic stroke, any stroke, death, and MACEs.

The performance of the CHADS_2_, CHA_2_DS_2_-VASc, ATRIA, and Essen stroke risk scores for the prediction of recurrent stroke was unsatisfactory in stroke patients with AF whereas the performance for the prediction of recurrent stroke was not MACEs or death was good. A new risk stratification scheme that is specific for secondary stroke prevention in the AF population is needed.

## Abstract 2

Supplemental Digital Content is available in the text

## 1 Introduction

A good prognostication for thromboembolic events in patients with atrial fibrillation (AF) is essential because AF is a significant cause of ischemic stroke with a 5-fold increased risk.^[1]^ Various clinical risk scoring systems for the thromboembolic risk in patients with AF were developed and validated, such as the CHADS_2_,^[2]^ CHA_2_DS_2_-VASc,^[3]^ and ATRIA scores.^[4]^ These scoring systems developed to predict thromboembolic risk and provide a guidance for decision for anticoagulation. Recent clinical practice guidelines are recommending these scoring systems as a guidance for decision of oral anticoagulant (OAC) use in AF patients.^[5,6]^ However, their performance has rarely been validated in stroke patients with AF, especially in real-world setting in the era of newer OAC.

We also analyzed the validity of the Essen stroke score to verify the validity of other scores in this study. The Essen stroke risk score, which was introduced in CAPRIE trial^[7]^ and validated using the data set of the European Stroke Prevention study II^[8]^ is used for prediction of recurrent stroke and combined cerebrovascular events in stroke patient without AF.^[9]^ Therefore, we assumed that the performance of Essen stroke risk score is lower than those of CHADS_2_, CHA_2_DS_2_-VASc, and ATRIA scores for the prediction of vascular events in patients with AF. If the assumption is not correct, then the exclusive value of CHADS_2_, CHA_2_DS_2_-VASc, and ATRIA scores for the prediction of vascular events in AF patients will be unacceptable.

Therefore, we investigated the validity and performance of each scoring system in predicting vascular events along with the incidence rate of vascular events using data obtained from the K-ATTENTION (Korean ATrial fibrillaTion EvaluatioN regisTry in Ischemic strOke patieNts) study.

## 2 Methods

### 2.1 Registry data sources

The K-ATTENTION study is a multicenter, cohort study by merging of prospectively collected stroke registries from 11 tertiary hospitals in 5 provinces of South Korea to investigate the diagnosis, treatment, and prognosis of acute stroke patients with AF in a real-world clinical setting. Details of the K-ATTENTION study was described in elsewhere.^[10]^ In brief, subjects over 20 years of age, diagnosed with acute ischemic stroke with AF within 7 days from the onset were included consecutively from each participating center between January 1, 2013, to December 31, 2015. Subjects who were not adequately screened for stroke and arrhythmia and without evidence of cerebral infarction on brain images were excluded. The study protocol was reviewed and approved by the institutional review board of each participating center. Because of the retrospective nature of this study, the ethical board of each center exempted written informed consent.

### 2.2 Clinical data collection

The K-ATTENTION study collects demographic data (age, sex), physical examinations, vascular risk factors, previous medication history, and vascular outcomes. Vascular outcomes included recurrent ischemic stroke, any stroke, death, and major adverse cerebrovascular and cardiovascular events (MACEs). MACE was defined as a composite of any stroke (ischemic or hemorrhagic), myocardial infarction and death. The 11 tertiary hospitals followed above definition for collect vascular outcomes. We obtained data including date, type, and causes of stroke recurrence, mortality from well-trained research nurses or neurology specialists at each hospital. If the patient did not visit hospital, clinical information was obtained from the patient or their care-givers by telephone interview or by review of medical records. As 1 site did not provide long-term vascular outcome data, all subjects in that site (n = 101) were excluded in this study. And, the censoring date was set at December 31, 2016 or the last date when the investigator had contact with the subject.

### 2.3 Data management and quality control

All data were collected and uploaded via a web-based electronic data capturing system. All investigators accessed this secure database system and registered mandatory variables related to the answer of primary objects. The collected data were monitored and audited by the quality control team.

### 2.4 The risk scoring systems

We calculated the CHADS_2_, CHA_2_DS_2_-VASc, ATRIA, and Essen stroke risk scores for each subject using the data thus obtained. The CHADS_2_ score includes congestive heart failure, hypertension, age ≥ 75 years, diabetes mellitus, prior stroke, or transient ischemic attack (2 points), resulting in a maximum score of 6 points. The CHA_2_DS_2_-Vasc score includes congestive heart failure, hypertension, age ≥ 75 years (2 points), diabetes mellitus, previous stroke, or transient ischemic attack (2 points), vascular disease, age 65 to 74 years, and sex (female), resulting in a maximum score of 9 points. The ATRIA score with the previous stroke includes age < 65 years (8 points), age 65 to 84 years (7 points), age ≥ 85 years (9 points), sex (female), congestive heart failure, hypertension, diabetes mellitus, proteinuria, estimated glomerular filtration rate <45 mL/min per 1.73 m^2^, or end-stage renal disease requiring renal replacement therapy, resulting in a maximum score of 15 points. The Essen stroke risk score includes age 65 to 75 years, age ≥ 75 years (2 points), hypertension, diabetes mellitus, myocardial infarction, prior stroke or transient ischemic attack, smoking, peripheral arterial disease, and other cardiovascular disease (except myocardial infarction and AF), resulting in a maximum score of 9 points.

Based on information in the registries, we calculated the CHADS_2_ score, CHA_2_DS_2_-VASc score, ATRIA score, and Essen stroke risk scores for all subjects at the time of admission for the stroke. Subjects with CHADS_2_ score ≥ 5 were merged into 1 category, and subjects with CHA_2_DS_2_-VASc score 2 and 3 were merged into 1 category, as were those with score ≥7; subjects with ATRIA score 7 and 8 were merged into 1 group, as well as those with score ≥12; subjects with Essen stroke risk score 0 and 1 were combined into 1 category, and so were those with score ≥5.

### 2.5 Statistical analysis

The data are expressed as mean value and standard deviation, for continuous data, and frequency and percentage for categorical data. For comparisons between the groups, we used Student *t* test and ANOVA for continuous data and Chi-squared and Fisher exact tests for categorical data. Using the Cox regression model, considering death as a competing risk, the predictive value of the CHADS_2_, CHA_2_DS_2_-VASc, ATRIA, and Essen stroke risk scores to the vascular outcome was investigated. Performance for the predictability for vascular outcomes was presented as overall C-index with 95% confidence interval and time-dependent C-index for each scoring system. A separate data management committee managed all data, and an external team performed statistical analyses. All statistical analyses were performed using SAS 9.4 (SAS Institute, NC) and STATA 13 (StataCorp, TX).

## 3 Results

### 3.1 Study population

A total of 3112 stroke with AF subjects (mean age 73.5 ± 0.2; 48.6% female) were included, after exclusion of 101 subjects without data on vascular events, among total 3213 subjects included in K-ATTENTION study. Mean duration of follow up was 1399.6 ± 15.8 days (95% confidence interval [CI] 1368.7–1430.5). The baseline characteristics of the subjects are shown in Table 1. A total of 2519 subjects were prescribed OAC after stroke for secondary stroke prevention, while 593 were not. Subjects without OAC treatment, compared to subjects with OAC treatment, were older (76.5 ± 0.4 vs 72.8 ± 0.2 years.), more often women (55.1% vs 47%), had higher initial National Institute of Health Stroke Scale score (13.6 ± 0.3 vs 8.3 ± 0.1), and higher modified Rankin Scale at 90 days (3.7 ± 0.1 vs 2.7 ± 0.0).

**Table 1:** Baseline Characteristics.

	OACs (–) (n = 593)	OACs (+) (n = 2519)	*P*	Total (n = 3112)
Age, yr	76.5 ± 0.4	72.8 ± 0.2	<.01	73.5 ± 0.2
>65	73 (12.3)	455 (18.1)		528 (17.0)
65–74	152 (25.6)	824 (32.7)		976 (31.4)
≥75	368 (62.1)	1240 (49.2)		1608 (51.7)
Sex, women	327 (55.1)	1335 (53.0)	<.01	1511 (48.6)
Risk factors				
Previous Stroke	207 (34.9)	839 (33.3)	.46	1046 (33.6)
Congestive heart failure	21 (3.5)	110 (4.4)	.37	131 (4.2)
Hypertension	417 (70.3)	1737 (69.0)	.52	2154 (69.2)
Diabetes mellitus	157 (26.5)	674 (26.8)	.89	831 (26.7)
Dyslipidemia	107 (18.0)	640 (25.4)	<.01	747 (24.0)
Coronary artery disease	68 (11.5)	332 (13.2)	.26	400 (12.9)
Peripheral artery disease	6 (1.0)	32 (1.3)	.61	38 (1.2)
Initial NIHSS	13.59 ± 0.3	8.28 ± 0.1	<.01	9.29 ± 0.1
mRS at 3 months	3.73 ± 0.1	2.65 ± 0.0	<.01	2.88 ± 0.0
Discharge medication				
Non-antiplatelet	346 (58.4)	1964 (78.0)		2310 (74.2)
Mono antiplatelet	178 (30.0)	474 (18.8)		652 (21.0)
Dual antiplatelet	69 (11.6)	81 (3.2)		150 (4.8)
Warfarin	0 (0)	1818 (72.2)		1818 (58.4)
NOAC	0 (0)	701 (27.8)		701 (22.5)

### 3.2 Distribution of CHADS_2_, CHA_2_DS_2_-VASc, ATRIA, and Essen stroke risk scores

The distribution of risk scores according to the various systems is shown in Table 2. The mean CHADS_2_, CHA_2_DS_2_-VASc, ATRIA, and Essen stroke risk scores were significantly different between the subjects with OAC treatment (3.4 ± 0.0, 4.8 ± 0.0, 9.1 ± 0.0, and 3.1 ± 0.0, respectively) and those without OAC treatment (3.6 ± 0.0, 5.1 ± 0.1, 9.4 ± 0.1, and 3.3 ± 0.1, respectively).

**Table 2:** Distribution of Subjects for Each Score Category in CHADS_2_, CHA_2_DS_2_-VASc, ATRIA, and Essen stroke risk scores, According to oral anticoagulation treatment.

	OACs (–), (n = 593) n (%)	OACs (+), (n = 2519) n (%)	Total (n = 3112) n (%)
CHADS_2_			
Score 2	74 (12.5)	431 (17.1)	505 (16.2)
Score 3	188 (31.7)	881 (35.0)	1069 (34.4)
Score 4	236 (39.8)	926 (36.8)	1162 (37.6)
Score ≥ 5	95 (16.0)	281 (11.2)	376 (12.1)
CHA_2_DS_2_-VASc			
Score 2 or 3	74 (12.5)	459 (18.2)	533 (17.1)
Score 4	106 (17.9)	526 (20.9)	632 (20.3)
Score 5	139 (23.4)	652 (25.9)	791 (25.4)
Score 6	193 (32.5)	608 (24.1)	801 (25.7)
Score ≥ 7	81 (13.7)	274 (10.9)	355 (11.4)
ATRIA			
Score 7 or 8	156 (26.5)	810 (32.8)	966 (31.6)
Score 9	172 (29.3)	829 (33.6)	1001 (32.8)
Score 10	133 (22.6)	477 (19.3)	610 (20.0)
Score 11	66 (11.2)	236 (9.6)	302 (9.9)
Score ≥ 12	61 (10.4)	114 (4.6)	175 (5.7)
Essen stroke			
Score 0 or 1	43 (7.3)	332 (13.2)	375 (12.1)
Score 2	114 (19.2)	479 (19.0)	593 (19.1)
Score 3	196 (33.1)	744 (29.5)	940 (30.2)
Score 4	144 (24.3)	597 (23.7)	741 (23.8)
Score ≥ 5	96 (16.2)	367 (14.6)	463 (14.9)

### 3.3 Association between vascular outcomes and CHADS_2_, CHA_2_DS_2_-VASc, ATRIA, and Essen stroke risk scores

Supplemental Table 1, presents the cumulative incidence rates for each outcome. The annualized incidence rates for all outcomes were higher in the non-OAC group than the OAC group.

**Figure 1:**
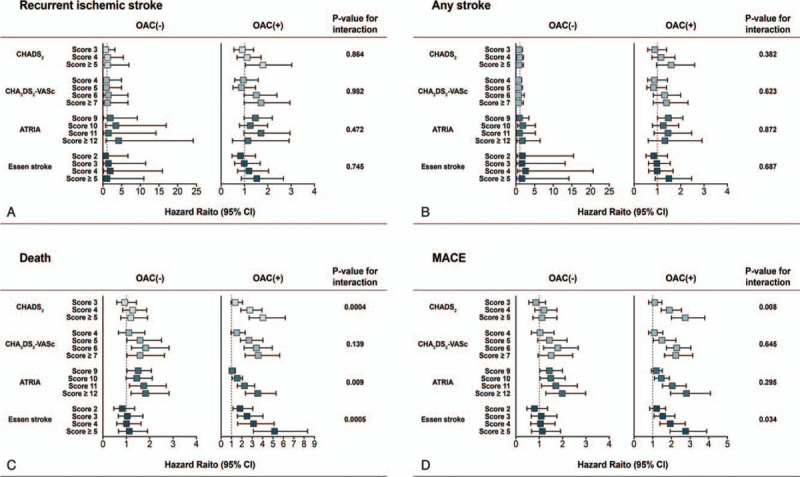
Forest plots for competing risk analysis showing hazard ratios of outcomes stratified by oral anticoagulant treatment history and risk scoring systems.

There was no significant association between the risk of recurrent ischemic stroke or any stroke with the 4 scores (Fig. 1 and Supplemental Table 2). The trend of negative association was consistent in both the OAC group and non-OAC group. However, the risks of death and MACE increased sequentially with the increase of each risk score. The association between risk scores and the risk of death or MACE was different between the OAC and non-OAC group. All 4 scores were predictive death and MACE in the OAC group. However, in the non-OAC group, the CHA_2_DS_2_-VASc score (*P* = .01) and the ATRIA score (*P* = .03) for death, and the CHA_2_DS_2_-VASc score (*P* = .01) and the ATRIA (*P* = .02) scores for MACE were predictive. OAC treatment had a significant interaction with the CHADS_2_, ATRIA, and Essen stroke risk scores in terms of predicting death (*P* < .01, *P* < .01, and *P* < .01, respectively) and with the CHADS_2_ and Essen stroke risk scores in terms of predicting MACE (*P* < .01 and *P* = .03, respectively).

**Figure 2:**
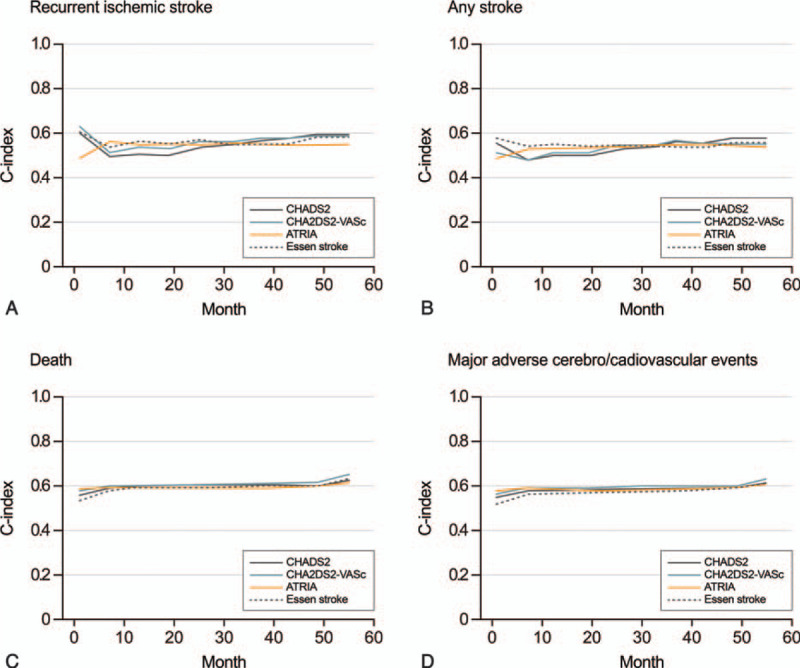
C-Statistics for the 4 scoring systems and vascular outcomes.

The overall performance of all scoring systems for the prediction of recurrent ischemic stroke, any stroke, death, and MACE were unsatisfactory (0.54–0.56 for recurrent stroke, 0.52–0.54 for any stroke, 0.58–0.60 for death, and 0.56–0.58 for MACE, respectively; Table 3). Figure 2, which shows the C-index of each scoring system according to the time points, revealed no sizable differences among the scoring systems. Each score in the OAC group showed higher performance (C-index: 0.59–0.63 for death and 0.58–0.60 for MACE; Supplemental Table 3, Supplemental Fig. 1 and 2) than that in the non-OAC group (C-index: 0.51–0.53 for death and 0.51–0.54 for MACE).

**Table 3:** Overall C-index for the 4 scoring systems and vascular outcomes.

	C-index (95% confidence interval)			
	CHADS_2_,	CHA_2_DS_2_-VASc	ATRIA	Essen stroke risk
Recurrent Ischemic stroke	0.54 (0.50–0.59)	0.56 (0.51–0.60)	0.55 (0.51–0.60)	0.55 (0.50–0.60)
Any stroke	0.53 (0.49–0.57)	0.52 (0.48–0.57)	0.54 (0.47–0.58)	0.53 (0.48–0.57)
Death	0.59 (0.60–0.62)	0.60 (0.58–0.62)	0.59 (0.56–0.61)	0.58 (0.56–0.60)
MACE	0.58 (0.57–0.60)	0.58 (0.56–0.60)	0.58 (0.57–0.60)	0.56 (0.54–0.59)

## 4 Discussion

In this study, we investigated the performance of the CHADS_2_, CHA_2_DS_2_-VASc, ATRIA, and Essen stroke risk scores in predicting recurrent ischemic stroke, any stroke, death, and MACE in stroke patient with AF, using a nationwide multicenter registry data. The overall performance of all the scoring systems for each outcome was unsatisfactory. In particular, these scoring systems showed poor performance in predicting stroke recurrence. Although these scores showed a better performance in the prediction of death and MACE, the CHADS_2_, CHA_2_DS_2_-VASc, ATRIA scores did not outperform the Essen stroke risk score. Besides, the performance of the CHADS_2_, ATRIA, and Essen stroke risk scores for death and MACE were different between the OAC-treated and the non-OAC-treated groups.

Previous studies revealed that the CHADS_2_, CHA_2_DS_2_-VASc, ATRIA scores performed reasonably well in the prediction of vascular events and mortality in patients with AF. ^[11–13]^ So, in clinical practice, the CHADS_2_, CHA_2_DS_2_-VASc, ATRIA scores have been commonly used for the stratification of future stroke risk in patients with AF. Moreover, current clinical practice guidelines recommend the use of OAC in AF patients with moderate to high risk of a thromboembolic event, based on the CHA2DS2-VASc score.^[6]^ However, the performance of the CHADS_2_, CHA_2_DS_2_-VASc, ATRIA scores in stroke patients with AF was unsatisfactory than our expectation. The initial validation studies for CHADS_2,_^[2]^ CHA_2_DS_2_-VASc,^[3]^ and ATRIA^[4]^ reported that C-statistics for predicting thromboembolic event were 0.82, 0.61, and 0.70, respectively. Recent meta-analyses reported somewhat lower C-statistics for each score than those in initial validation studies (0.58–0.70 for CHA_2_DS_2_-VASc and 0.63–0.69 for ATRIA scores).^[14,15]^ However, our results showed even more lower performance than the results of meta-analysis (0.54–0.56 for ischemic stroke, 0.58–0.60 for death, and 0.56–0.58 for MACE, respectively). Considering that the decision whether to use OAC or not depends on the CHA_2_DS_2_-VASc score, this predictive value for recurrent stroke seems too low to be acceptable.

The condition of the subjects or geographical region could affect the performance of the CHADS_2_ and CHA_2_DS_2_-VASc scores. The subject of this study were acute stroke patients who are at a higher risk of death and co-morbidity, that can make the performance of the scores weaken. In the Asian population, the performance of the scores was lower than the non-Asian population. A Chinese study reported that the C-statistics of the CHADS_2_ and CHA_2_DS_2_-VASc scores in the China National Stroke Registry were 0.53 to 0.55 for predicting recurrent ischemic stroke, and 0.53 to 0.57 for predicting death in ischemic stroke patients with AF.^[16]^

In contrast to their poor performance for recurrent ischemic stroke or any stroke, all scores showed moderate performance for death and MACE. Previous studies on stroke patients with AF also reported that a high CHA_2_DS_2_-VASc score reflected poor short-term outcome, and higher CHADS_2_ and CHA_2_DS_2_-VASc scores were associated with severe stroke and a worse clinical course.^[17,18]^ Based on another Korean Study, high-risk CHADS_2_ scores were associated with worse neurological outcomes at discharge, and increased long-term mortality, especially due to vascular causes in stroke patients with AF.^[19]^ Our results are in agreement with these previous studies.

Another interesting finding was that OAC treatment had considerable influence on the performance of each scoring system, especially for the prediction of death or MACE. The performance of every score, except the CHA_2_DS_2_-VASc, improved when the subjects were confined to the OAC-treated group. In contrast, the performance of every score was unsatisfactory in the non-OAC-treated group. In clinical practice, the decision to initiate OAC in acute stroke patients can be influenced not only by the risk of stroke or thromboembolism based on stratification by risk scores, but also by other factors such as medical comorbidity, neurological status, or the risk of intracerebral hemorrhage assessed by neuroimaging. Therefore, application of these scores to non-OAC-treated patients seems inappropriate, because the primary target of these scores was to predict stroke or thromboembolic risk in AF patients precisely. However, their performance, in this study, was better for death and MACE.

The 4 scores shared a similar performance in this study because they all share 4 main items, including age, hypertension, diabetes, and previous stroke. Considering that all subjects in this study were acute stroke patients, they had high-risk of stroke or thromboembolism. For example, the CHA_2_DS_2_-VASc score outperforms CHADS_2_ in discriminating very low-risk subjects.^[5]^ However, such discriminative power is not useful in this study, because all subjects were rated at least 2 points by the CHADS_2_ or CHA_2_DS_2_-VASc scores. Furthermore, CHADS_2_ and CHA_2_DS_2_-VASc scores did not outperform the Essen stroke risk score. Vice versa, it was reported that, using the Danish Stroke Registry, the C-statistics of both the Essen and CHA_2_DS_2_-VASc scores for predicting recurrent stroke in patients without AF was 0.54 to 0.56.^[20]^ In the Athens Stroke Registry, the CHADS_2_ and the CHA_2_DS_2_-VASc scores were predictive of recurrent stroke and death in non-AF stroke patients.^[9]^ To summarize, all scoring systems, including the Essen stroke risk score, are similarly predictive of the risk of stroke or other vascular events, irrespective of AF, implying that these scoring systems are not specific for AF patients. Therefore, a newer scoring system is required, truly specific for AF patients. Regarding the limitations of these scoring systems, a recent effort to find AF-specific biomarkers that can predict vascular events, such as fibrin clot permeability,^[21]^ N-terminal pro B-type natriuretic peptide,^[22]^ Group/Differentiation Factor-15 (GDF-15),^[23]^ or free fatty acid,^[24]^ could shed light on risk stratification in stroke patients with AF.

### 4.1 Strengths and limitations

Several limitations pertain to this study. First, all study subjects were ethnically Korean requesting a cautious generalization of the results. Second, the details of oral anticoagulation therapy in individual subjects, such as the type and initiation of OAC used, and the time-in-therapeutic range in the case of treatment with vitamin K antagonist, were not considered. Third, we used baseline risk scores and did not reassess the risk scoring systems every year. A previous study reported that in AF patients, stroke risk as assessed by the CHA_2_DS_2_-VASc score is dynamic, and changes over time.^[25]^ However, the primary purpose of this study was to validate the performance of the scoring systems measured at baseline. Finally, the subjects with the previous history of stroke could have a higher risk for recurrent vascular events than those who have single events. In this study, weighting on the previous history of stroke before index event or risk scoring system before index events could improve the prediction for the vascular outcome. However, models using risk scores being set before the index stroke (not counting score for index stroke event) failed to improve c-statistics for the vascular events in comparison with the model using risk scores being set after index stroke (not presented data on the results section).

Despite these limitations, this study is based on one of the largest registries for stroke patients with AF in the Asian population. Using this study, we can raise the issue of the validity of current risk scoring systems in acute stroke patients with AF.

## 5 Conclusion

We found that, in a real-world dataset, the CHADS_2_ score, CHA_2_DS_2_-VASc score, ATRIA score, and Essen stroke risk score have limited value for the prediction of recurrent stroke in patients with AF. These scoring systems are valid for predicting death and MACE in stroke patients with AF. However, their validity depends on patient characteristics, such as OAC use. Therefore, a new risk stratification scheme that is specific for the AF population in secondary stroke prevention is needed.

## Author contributions

**Conceptualization:** Tae-Jin Song, Bum Joon Kim, Sung Hyuk Heo, Jin-Man Jung, Kyung-Mi Oh, Chi Kyung Kim, Sungwook Yu, Kwang Yeol Park, Jeong-Min Kim, Jong-Ho Park, Jay Chol Choi, Man-Seok Park, Joon-Tae Kim, Yang-Ha Hwang, Jong-Won Chung, Oh Young Bang, Geong-Moon Kim, Yong-Jae Kim, Woo-Keun Seo.

**Data curation:** Tae-Jin Song.

**Formal analysis:** Inwu Yu, Seonwoo Kim, Sook young Woo, Hyun Cho.

**Investigation:** Inwu Yu, Tae-Jin Song, Bum Joon Kim, Sung Hyuk Heo, Jin-Man Jung, Kyung-Mi Oh, Chi Kyung Kim, Sungwook Yu, Kwang Yeol Park, Jeong-Min Kim, Jong-Ho Park, Jay Chol Choi, Man-Seok Park, Joon-Tae Kim, Yang-Ha Hwang, Jong-Won Chung, Oh Young Bang, Geong-Moon Kim, Yong-Jae Kim, Woo-Keun Seo.

**Methodology:** Sung Hyuk Heo, Jin-Man Jung, Kyung-Mi Oh, Chi Kyung Kim, Sungwook Yu, Kwang Yeol Park, Jeong-Min Kim, Jong-Ho Park, Jay Chol Choi, Man-Seok Park, Joon-Tae Kim, Yang-Ha Hwang, Jong-Won Chung, Oh Young Bang, Geong-Moon Kim, Yong-Jae Kim, Seonwoo Kim, Sook young Woo, Hyun Cho, Woo-Keun Seo.

**Project administration:** Woo-Keun Seo.

**Resources:** Sungwook Yu, Kwang Yeol Park, Jeong-Min Kim, Jong-Ho Park, Man-Seok Park, Joon-Tae Kim, Yang-Ha Hwang, Jong-Won Chung, Oh Young Bang, Geong-Moon Kim, Yong-Jae Kim.

**Supervision:** Woo-Keun Seo.

**Visualization:** Seonwoo Kim, Sook young Woo, Hyun Cho.

**Writing – original draft:** Inwu Yu.

**Writing – review & editing:** Tae-Jin Song, Bum Joon Kim, Sung Hyuk Heo, Jin-Man Jung, Kyung-Mi Oh, Chi Kyung Kim, Sungwook Yu, Kwang Yeol Park, Jeong-Min Kim, Jong-Ho Park, Jay Chol Choi, Man-Seok Park, Joon-Tae Kim, Yang-Ha Hwang, Jong-Won Chung, Oh Young Bang, Geong-Moon Kim, Yong-Jae Kim, Seonwoo Kim, Sook young Woo, Hyun Cho, Woo-Keun Seo.

## Supplementary Material

Supplemental Digital Content

## Supplementary Material

Supplemental Digital Content

## Supplementary Material

Supplemental Digital Content

## Supplementary Material

Supplemental Digital Content

## Supplementary Material

Supplemental Digital Content
