# Supplementary material for: CHADS2, CHA2DS2-VASc, ATRIA, and Essen stroke risk scores in stroke with atrial fibrillation: A nationwide multicenter registry study
Source: Medicine (Baltimore). 2021 Jan 22;100(3):e24000. doi: 10.1097/MD.0000000000024000 (PMC7837865; doi:10.1097/MD.0000000000024000)

**Supplemental Figure 1. C-Statistics for the four scoring systems and vascular outcomes of non-OAC treated group**


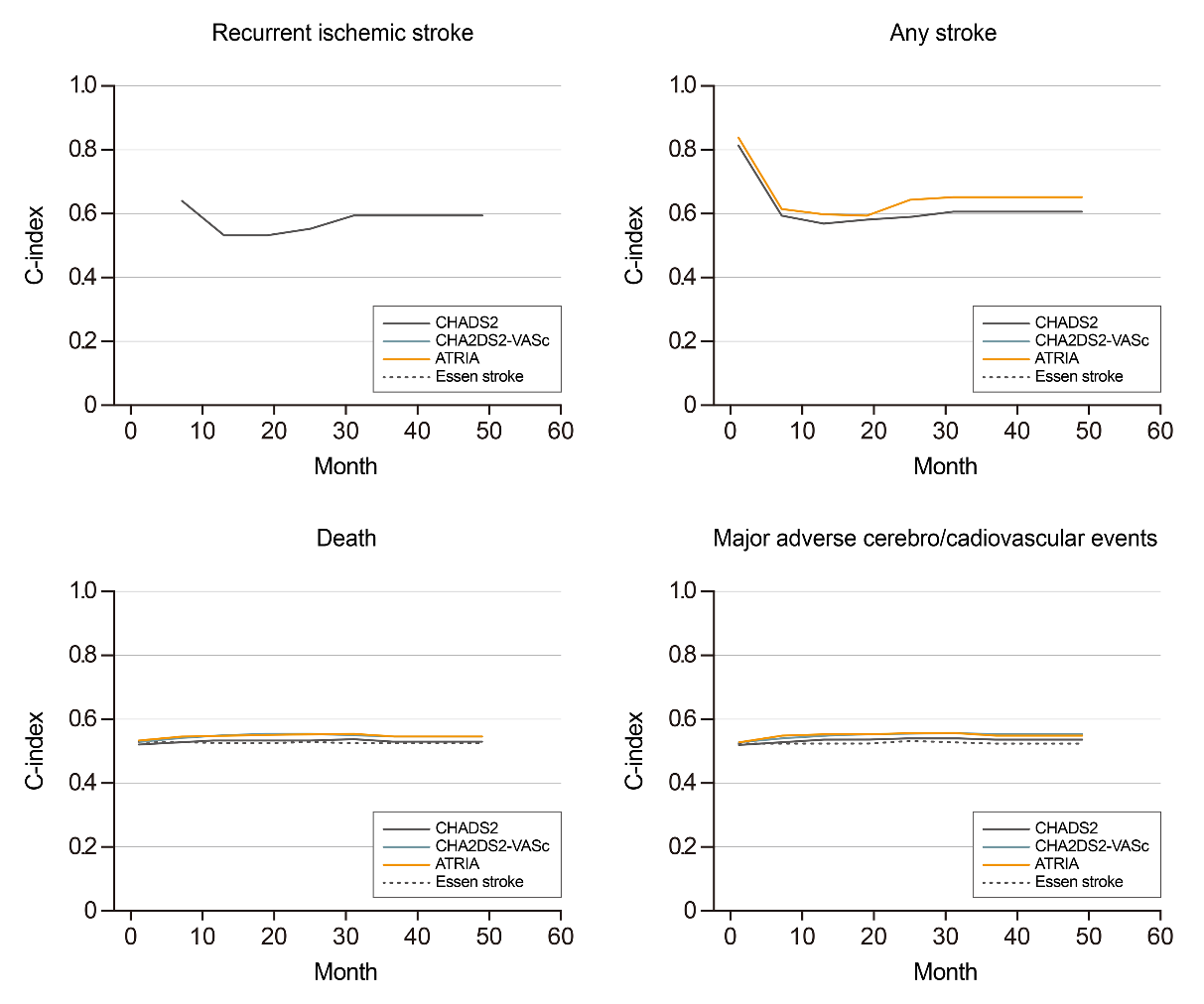

Supplement: Supplemental Digital Content [file medi-100-e24000-s001.docx]
