# Supplementary material for: CHADS2, CHA2DS2-VASc, ATRIA, and Essen stroke risk scores in stroke with atrial fibrillation: A nationwide multicenter registry study
Source: Medicine (Baltimore). 2021 Jan 22;100(3):e24000. doi: 10.1097/MD.0000000000024000 (PMC7837865; doi:10.1097/MD.0000000000024000)
Supplement: Supplemental Digital Content [file medi-100-e24000-s003.docx]

**Supplemental Table 1. The annualized and the cumulative incidence rate for each vascular outcome**

|  | Annual incidence rate | Cumulative incidence rate, % (95% confidence interval) | | | |
| --- | --- | --- | --- | --- | --- |
|  |  | 30 days | 90 days | 1 year | 2 years |
| Recurrent Ischemic stroke |  |  |  |  |  |
| Overall | 3.6 (3.1 - 4.2) | 0.5 (0.3-0.9) | 1.6 (1.2-2.2) | 3.8 (3.1-4.6) | 5.7 (4.7-6.7) |
| Non-OAC | 4.6 (2.8 - 7.6) | 0.2 (<0.1-1.1) | 0.9 (0.3-2.2) | 3.0 (1.5-5.3) | 7.0 (3.8-11.5) |
| OAC | 3.6 (3.0 - 4.2) | 0.6 (0.4-1.0) | 1.8 (1.3-2.4) | 4.0 (3.3-4.9) | 5.8 (4.8-6.9) |
| Any stroke |  |  |  |  |  |
| Overall | 4.5 (3.9 - 5.1) | 1.0 (0.7-1.5) | 2.3 (1.8-2.9) | 4.9 (4.1-5.8) | 7.1 (6.1-8.2) |
| Non-OAC | 6.7 (4.4 -10.2) | 0.8 (0.3-1.9) | 2.0 (1.0-3.6) | 4.3 (2.5-6.8) | 8.6 (5.2-13.1) |
| OAC | 4.3 (3.7 – 5.0) | 1.1 (0.7-1.6) | 2.4 (1.8-3.1) | 5.1 (4.2-6.1) | 7.2 (6.1-8.4) |
| Death |  |  |  |  |  |
| Overall | 12.3 (11.3 - 13.3) | 8.0 (7.0-9.0) | 11.0 (9.9-12.1) | 17.9 (16.5-19.4) | 21.1 (19.5-22.7) |
| Non-OAC | 75.8 (67.0 - 85.7) | 33.0 (29.1-37.0) | 38.7 (34.5-42.9) | 50.3 (45.5-54.9) | 53.2 (48.0-58.2) |
| OAC | 7.4 (6.7 - 8.3) | 2.35 (1.8-3.0) | 4.9 (4.0-5.8) | 11.1 (9.8-12.5) | 14.4 (12.9-16.0) |
| MACE |  |  |  |  |  |
| Overall | 16.5 (15.3 - 17.7) | 9.9 (8.81-11.0) | 13.7 (12.5-15.0) | 22.1 (20.5-23.7) | 26.9 (25.1-28.7) |
| Non-OAC | 82.1 (72.8 - 92.5) | 34.8 (30.8-38.8) | 40.5 (36.3-44.8) | 54.0 (49.1-58.7) | 58.9 (53.1-64.2) |
| OAC | 11.4 (10.4 - 12.4) | 4.3 (3.5-5.1) | 7.9 (6.8-9.0) | 15.45 (14.0-17.0) | 20.5 (18.7-22.3) |
