# Supplementary material for: CHADS2, CHA2DS2-VASc, ATRIA, and Essen stroke risk scores in stroke with atrial fibrillation: A nationwide multicenter registry study
Source: Medicine (Baltimore). 2021 Jan 22;100(3):e24000. doi: 10.1097/MD.0000000000024000 (PMC7837865; doi:10.1097/MD.0000000000024000)
Supplement: Supplemental Digital Content [file medi-100-e24000-s004.docx]

**Supplemental Table 2. Competing risk analysis and subgroup analysis showing hazard ratios of recurrent ischemic stroke stratified by OAC treatment and risk scoring systems**

|  |  | Non-OAC treated group | |  | OAC treated group | |  |  |  |
| --- | --- | --- | --- | --- | --- | --- | --- | --- | --- |
|  |  | HR | 95% CI |  | HR | 95% CI | p^*^ | p^†^ | p^‡^ |
| Recurrent ischemic stroke | | | | | | | | | |
| CHADS_2_ | Score 2 | 1 |  |  | 1 |  | 0.74 | 0.52 | 0.86 |
|  | Score 3 | 0.58 | 0.09–3.29 |  | 0.85 | 0.53–1.37 |  |  |  |
|  | Score 4 | 1.16 | 0.24–5.52 |  | 1.07 | 0.68–1.70 |  |  |  |
|  | Score ≥ 5 | 1.19 | 0.20–6.90 |  | 1.75 | 1.02–3.01 |  |  |  |
| CHA_2_DS_2_–VASc | Score 2 or 3 | 1 |  |  | 1 |  | 0.91 | 0.52 | 0.98 |
|  | Score 4 | 0.70 | 0.10–4.87 |  | 0.94 | 0.55–1.58 |  |  |  |
|  | Score 5 | 0.82 | 0.14–4.91 |  | 0.88 | 0.53–1.46 |  |  |  |
|  | Score 6 | 1.38 | 0.28–6.76 |  | 1.50 | 0.93–2.40 |  |  |  |
|  | Score ≥7 | 0.99 | 0.14–6.88 |  | 1.69 | 0.97–2.94 |  |  |  |
| ATRIA | Score 7 or 8 | 1 |  |  | 1 |  | 0.44 | 0.14 | 0.47 |
|  | Score 9 | 1.73 | 0.32–9.37 |  | 1.45 | 0.97–2.16 |  |  |  |
|  | Score 10 | 3.35 | 0.65–17.12 |  | 1.24 | 0.77–1.99 |  |  |  |
|  | Score 11 | 1.28 | 0.12–14.28 |  | 1.68 | 0.95–2.96 |  |  |  |
|  | Score ≥ 12 | 4.05 | 0.68–24.14 |  | 1.15 | 0.46–2.89 |  |  |  |
| Essen stroke | Score 0 or 1 | 1 |  |  | 1 |  | 0.67 | 0.46 | 0.75 |
|  | Score 2 | 0.43 | 0.03–6.75 |  | 0.80 | 0.44–1.44 |  |  |  |
|  | Score 3 | 1.36 | 0.16–11.59 |  | 0.96 | 0.57–1.64 |  |  |  |
|  | Score 4 | 1.93 | 0.23–15.96 |  | 1.18 | 0.69–2.00 |  |  |  |
|  | Score ≥ 5 | >0.99 | 0.09–10.84 |  | 1.50 | 0.85–2.64 |  |  |  |
| Any stroke | | | | | | | | | |
| CHADS_2_ | Score 2 | 1 |  |  | 1 |  | 0.43 | 0.56 | 0.38 |
|  | Score 3 | 0.36 | 0.11-1.26 |  | 0.88 | 0.57-1.36 |  |  |  |
|  | Score 4 | 0.58 | 0.19-1.74 |  | 1.15 | 0.76-1.74 |  |  |  |
|  | Score ≥ 5 | 0.45 | 0.11-1.87 |  | 1.57 | 0.95-2.61 |  |  |  |
| CHA_2_DS_2_–VASc | Score 2 or 3 | 1 |  |  | 1 |  | 0.58 | 0.69 | 0.62 |
|  | Score 4 | 0.41 | 0.10-1.71 |  | 0.90 | 0.57-1.44 |  |  |  |
|  | Score 5 | 0.43 | 0.11-1.61 |  | 0.87 | 0.55-1.36 |  |  |  |
|  | Score 6 | 0.72 | 0.23-2.19 |  | 1.29 | 0.84-1.98 |  |  |  |
|  | Score ≥ 7 | 0.38 | 0.08-1.93 |  | 1.39 | 0.83-2.32 |  |  |  |
| ATRIA | Score 7 or 8 | 1 |  |  | 1 |  | 0.90 | 0.46 | 0.87 |
|  | Score 9 | 1.04 | 0.32-3.40 |  | 1.46 | 1.01-2.09 |  |  |  |
|  | Score 10 | 1.59 | 0.48-5.21 |  | 1.23 | 0.80-1.90 |  |  |  |
|  | Score 11 | 1.01 | 0.20-5.24 |  | 1.44 | 0.84-2.47 |  |  |  |
|  | Score ≥ 12 | 1.58 | 0.38-6.61 |  | 1.30 | 0.59-2.87 |  |  |  |
| Essen stroke | Score 0 or 1 | 1 |  |  | 1 |  | 0.82 | 0.33 | 0.69 |
|  | Score 2 | 1.74 | 0.20-15.46 |  | 0.83 | 0.49-1.41 |  |  |  |
|  | Score 3 | 1.62 | 0.20-13.30 |  | 0.96 | 0.60-1.55 |  |  |  |
|  | Score 4 | 2.60 | 0.33-20.60 |  | 1.01 | 0.62-1.65 |  |  |  |
|  | Score ≥ 5 | 1.50 | 0.16-14.18 |  | 1.49 | 0.89-2.49 |  |  |  |
| Death | | | | | | | | | |
| CHADS_2_ | Score 2 | 1 |  |  | 1 |  | 0.19 | <0.001 | <0.01 |
|  | Score 3 | 0.92 | 0.60-1.40 |  | 1.32 | 0.87-2.00 |  |  |  |
|  | Score 4 | 1.25 | 0.84-1.88 |  | 2.66 | 1.81-3.91 |  |  |  |
|  | Score ≥ 5 | 1.19 | 0.74-1.90 |  | 4.00 | 2.60-6.16 |  |  |  |
| CHA_2_DS_2_–VASc | Score 2 or 3 | 1 |  |  | 1 |  | 0.01 | <0.01 | 0.14 |
|  | Score 4 | 1.10 | 0.67-1.78 |  | 1.40 | 0.88-2.23 |  |  |  |
|  | Score 5 | 1.58 | 1.01-2.48 |  | 2.64 | 1.76-3.97 |  |  |  |
|  | Score 6 | 1.84 | 1.20-2.84 |  | 3.28 | 2.19-4.93 |  |  |  |
|  | Score ≥ 7 | 1.60 | 0.97-2.64 |  | 3.53 | 2.24-5.55 |  |  |  |
| ATRIA | Score 7 or 8 | 1 |  |  | 1 |  | 0.03 | <0.01 | <0.01 |
|  | Score 9 | 1.47 | 1.05-2.07 |  | 1.00 | 0.74-1.34 |  |  |  |
|  | Score 10 | 1.44 | 0.99-2.09 |  | 1.49 | 1.09-2.04 |  |  |  |
|  | Score 11 | 1.74 | 1.12-2.70 |  | 2.18 | 1.53-3.10 |  |  |  |
|  | Score ≥ 12 | 1.83 | 1.18-2.84 |  | 3.45 | 2.28-5.23 |  |  |  |
| Essen stroke | Score 0 or 1 | 1 |  |  | 1 |  | 0.55 | <0.01 | <0.01 |
|  | Score 2 | 0.79 | 0.47-1.35 |  | 1.75 | 1.03-2.99 |  |  |  |
|  | Score 3 | 1.04 | 0.64-1.70 |  | 2.39 | 1.45-3.92 |  |  |  |
|  | Score 4 | 0.99 | 0.60-1.64 |  | 3.07 | 1.87-5.04 |  |  |  |
|  | Score ≥ 5 | 1.13 | 0.67-1.91 |  | 5.05 | 3.07-8.32 |  |  |  |
| MACE | | | | | | | | | |
| CHADS_2_ | Score 2 | 1 |  |  | 1 |  | 0.11 | <0.01 | <0.01 |
|  | Score 3 | 0.84 | 0.56-1.26 |  | 1.08 | 0.80-1.46 |  |  |  |
|  | Score 4 | 1.19 | 0.81-1.75 |  | 1.89 | 1.42-2.51 |  |  |  |
|  | Score ≥ 5 | 1.12 | 0.72-1.76 |  | 2.73 | 1.96-3.80 |  |  |  |
| CHA_2_DS_2_–VASc | Score 2 or 3 | 1 |  |  | 1 |  | 0.01 | <0.01 | 0.65 |
|  | Score 4 | 1.02 | 0.64-1.63 |  | 1.07 | 0.77-1.50 |  |  |  |
|  | Score 5 | 1.45 | 0.94-2.23 |  | 1.64 | 1.22-2.22 |  |  |  |
|  | Score 6 | 1.78 | 1.18-2.69 |  | 2.26 | 1.69-3.03 |  |  |  |
|  | Score ≥ 7 | 1.51 | 0.93-2.44 |  | 2.22 | 1.58-3.14 |  |  |  |
| ATRIA | Score 7 or 8 | 1 |  |  | 1 |  | 0.02 | <0.01 | 0.30 |
|  | Score 9 | 1.43 | 1.03-1.99 |  | 1.17 | 0.92-1.47 |  |  |  |
|  | Score 10 | 1.47 | 1.03-2.11 |  | 1.45 | 1.12-1.88 |  |  |  |
|  | Score 11 | 1.73 | 1.12-2.65 |  | 2.04 | 1.51-2.76 |  |  |  |
|  | Score ≥ 12 | 1.97 | 1.30-3.01 |  | 2.79 | 1.91-4.07 |  |  |  |
| Essen stroke | Score 0 or 1 | 1 |  |  | 1 |  | 0.421 | <.0001 | 0.034 |
|  | Score 2 | 0.793 | 0.471-1.333 |  | 1.176 | 0.809-1.709 |  |  |  |
|  | Score 3 | 1.097 | 0.682-1.766 |  | 1.522 | 1.082-2.141 |  |  |  |
|  | Score 4 | 1.039 | 0.638-1.693 |  | 1.932 | 1.373-2.720 |  |  |  |
|  | Score ≥ 5 | 1.142 | 0.682-1.910 |  | 2.713 | 1.904-3.866 |  |  |  |

Cox-regression was used for predicting vascular event, considering death as the competing risk

CI, confidence interval.

^*^ p-value for the main effect in OAC (-); ^†^ p-value for main effect of OAC (+); ^‡^ p-value for the interaction
