# Supplementary material for: CHADS2, CHA2DS2-VASc, ATRIA, and Essen stroke risk scores in stroke with atrial fibrillation: A nationwide multicenter registry study
Source: Medicine (Baltimore). 2021 Jan 22;100(3):e24000. doi: 10.1097/MD.0000000000024000 (PMC7837865; doi:10.1097/MD.0000000000024000)
Supplement: Supplemental Digital Content [file medi-100-e24000-s005.docx]

**Supplemental Table 3. Overall C-Statistics according to OAC treatment history for the four scoring systems and vascular outcomes**

|  | C-index (95% confidence interval) | | | |
| --- | --- | --- | --- | --- |
| Non-OAC treated group |  |  |  |  |
| Recurrent Ischemic stroke | 0.60 (0.44-0.68) | NA | NA | NA |
| Any stroke | 0.55 (0.43-0.68) | NA | 0.56 (0.41-0.70) | NA |
| Death | 0.52 (0.48-0.55) | 0.53 (0.50-0.57) | 0.53 (0.50-0.5) | 0.51 (0.47-0.55) |
| MACE | 0.52 (0.48-0.55) | 0.53 (0.50-0.57) | 0.54 (0.50-0.57) | 0.51 (0.47-0.55) |
| OAC treated group |  |  |  |  |
| Recurrent Ischemic stroke | 0.54 (0.49-0.59) | 0.56 (0.51-0.60) | 0.54 (0.50-0.59) | 0.54 (0.49-0.59) |
| Any stroke | 0.53 (0.49-0.57) | 0.53 (0.48-0.57) | 0.53 (0.49-0.58) | 0.53 (0.48-0.57) |
| Death | 0.63 (0.60-0.66) | 0.62 (0.59-0.65) | 0.59 (0.57-0.63) | 0.62 (0.59-0.65) |
| MACE | 0.60 (0.57-0.62) | 0.59 (0.56-0.62) | 0.58 (0.55-0.60) | 0.59 (0.56-0.61) |

See the footnote of Table I for abbreviations.

NA, not applicable

We could not analyze the c-index of CHA_2_DS_2_-VASc, ATRIA and Essen stroke risk scores because no events of recurrent ischemic stroke in non-OAC treated group. For same reason, we could not analyze the c-index of CHA_2_DS_2_-VASc for any stroke in non-OAC treated group.
